# Comparative efficacy of uncut Roux-en-Y and Billroth II anastomosis in gastrointestinal reconstruction following laparoscopic radical gastrectomy for distal gastric cancer

**DOI:** 10.1097/MD.0000000000037037

**Published:** 2023-02-02

**Authors:** Bufei Zhao, Zhun Yu, Ting Hu

**Affiliations:** a Department of Hepatopancreatobiliary, Affiliated Hospital of BeiHua University, Jilin, Jilin Province, China; b Department of Cardiology, The 3rd Affiliated Hospital of CCUCM, Changchun, Jilin Province, China; c Outpatient office, The First Affiliated Hospital of Changchun University of Chinese Medicine, Changchun, Jilin Province, China.

**Keywords:** Billroth II anastomosis, distal gastric cancer, gastrointestinal reconstruction, laparoscopic D2 gastrectomy, uncut Roux-en-Y anastomosis

## Abstract

This study retrospectively analyzed the clinical efficacy of Uncut Roux-en-Y and Billroth II anastomoses in gastrointestinal reconstruction following laparoscopic D2 radical gastrectomy for distal gastric cancer. The primary objective was to compare the postoperative outcomes, including quality of life and complication rates, between the 2 surgical techniques. One hundred patients diagnosed with distal gastric cancer were enrolled between June 2020 and May 2023. Patients underwent laparoscopic D2 gastrectomy and were categorized into either the Uncut Roux-en-Y or Billroth II anastomosis groups based on the technique used for gastrointestinal reconstruction. The inclusion and exclusion criteria were strictly followed. Surgical parameters, quality of life assessed using the Visick grading index, and postoperative complications were also evaluated. Statistical analyses were performed using SPSS version 27.0. The groups were comparable in terms of demographic and baseline clinical parameters. The Uncut Roux-en-Y group had a significantly longer duration of surgery (*P* < .001). However, there were no statistically significant differences in other surgical parameters. According to the Visick grading index, patients in the Uncut Roux-en-Y group reported a significantly better quality of life than those in the Billroth II group (*P* < .05). Additionally, Uncut Roux-en-Y was associated with a significantly lower incidence of dumping syndrome and bile reflux (*P* < .05). Although Uncut Roux-en-Y anastomosis requires longer surgical time, it offers significant advantages in terms of postoperative quality of life and reduced rates of dumping syndrome and bile reflux. Our findings suggest that Uncut Roux-en-Y may be a superior option for gastrointestinal reconstruction after laparoscopic D2 gastrectomy for distal gastric cancer.

## 1. Introduction

Gastric cancer poses a significant global health burden, ranking as the fifth most prevalent form of cancer and third leading cause of cancer-related mortality worldwide. Despite substantial advancements in diagnostic methods and therapeutic interventions, the overall prognosis of this malignancy remains unclear and is often attributed to late-stage diagnosis and the inherently aggressive nature of the disease.^[1,2]^ Among the various subtypes of gastric cancer, distal gastric cancer, notably tumors localized to the lesser curvature of the gastric antrum, is particularly common. The primary treatment modality for distal gastric cancer with curative intent is laparoscopic radical gastrectomy, which has been shown to yield promising clinical results.^[3,4]^ However, this surgical approach is not devoid of challenges, as it substantially modifies the anatomical and physiological architecture of the stomach. These alterations frequently lead to a range of pathophysiological changes and digestive symptoms postoperatively, thereby affecting the quality of life of patients.

Reconstruction of the gastrointestinal tract following gastrectomy plays an indispensable role in patient outcomes, both in terms of quality of life and efficacy of tumor eradication. Common techniques for reconstructive surgery following gastrectomy include the Billroth I, Billroth II, and traditional Roux-en-Y approaches.^[5]^ Each of these techniques has its merits and drawbacks, and the choice of method has a direct bearing on the postoperative patient well-being. The Billroth II method is recognized for its lower propensity for anastomotic leaks due to reduced tension at the anastomotic junction.^[6,7]^ Nonetheless, this form of anastomosis introduces several complications owing to the alteration of physiological pathways for the flow of gastric juice, bile, and pancreatic secretions. These alterations can result in detrimental complications such as alkaline reflux. This technique, differing from the traditional Roux-en-Y by preserving a portion of the small intestine uncut, has been increasingly recognized for maintaining near-physiological digestive flow while potentially reducing morbidity.^[8,9]^ Given the significant repercussions that these surgical choices can have on patient outcomes, rigorous evaluation and comparison of these reconstruction techniques is of utmost importance.

Therefore, the primary objective of this study was to perform a comparative analysis of the efficacy of the Uncut Roux-en-Y and Billroth II anastomosis techniques in gastrointestinal reconstruction following laparoscopic radical gastrectomy for distal gastric cancer. This comparison aimed to offer critical insights into the merits and limitations of these commonly employed methods, with a particular focus on postoperative quality of life and efficacy of tumor eradication.

## 2. Materials and methods

### 2.1. Study design

This study was a retrospective investigation carried out at the Affiliated Hospital of Beijing Hua University, encompassing patient data collected from June 2020 to May 2023. The primary focus of this research was on individuals diagnosed with distal gastric cancer who had undergone laparoscopic D2 radical gastrectomy and subsequent gastrointestinal reconstruction. Patients were stratified into 2 groups based on the type of anastomosis performed: Uncut Roux-en-Y and Billroth II.

*Patients were included in the study based on the following criteria:*

1. The diagnosis of distal gastric cancer was confirmed using a comprehensive set of diagnostic measures, including gastroscopy, biopsy, upper gastrointestinal barium radiography, and histopathological examinations.
2. Underwent laparoscopic D2 radical gastrectomy as the chosen treatment modality for distal gastric cancer.
3. No prior history of radiotherapy, chemotherapy, traditional Chinese medical treatment, or targeted therapy.
4. Availability of complete clinical and follow-up data.

*Patients were excluded from the study for the following reasons:*

1. Presence of other malignant tumors in addition to gastric cancer.
2. Diagnosis of metastatic gastric cancer.
3. The occurrence of acute complications related to gastric cancer, such as perforation or bleeding, that required emergency surgery prior to the laparoscopic procedure.
4. Requirement for conversion from laparoscopic to open surgery during the procedure.
5. Intraoperative findings indicated involvement of other organs in gastric cancer, necessitating combined organ resection.

**Figure 1.**
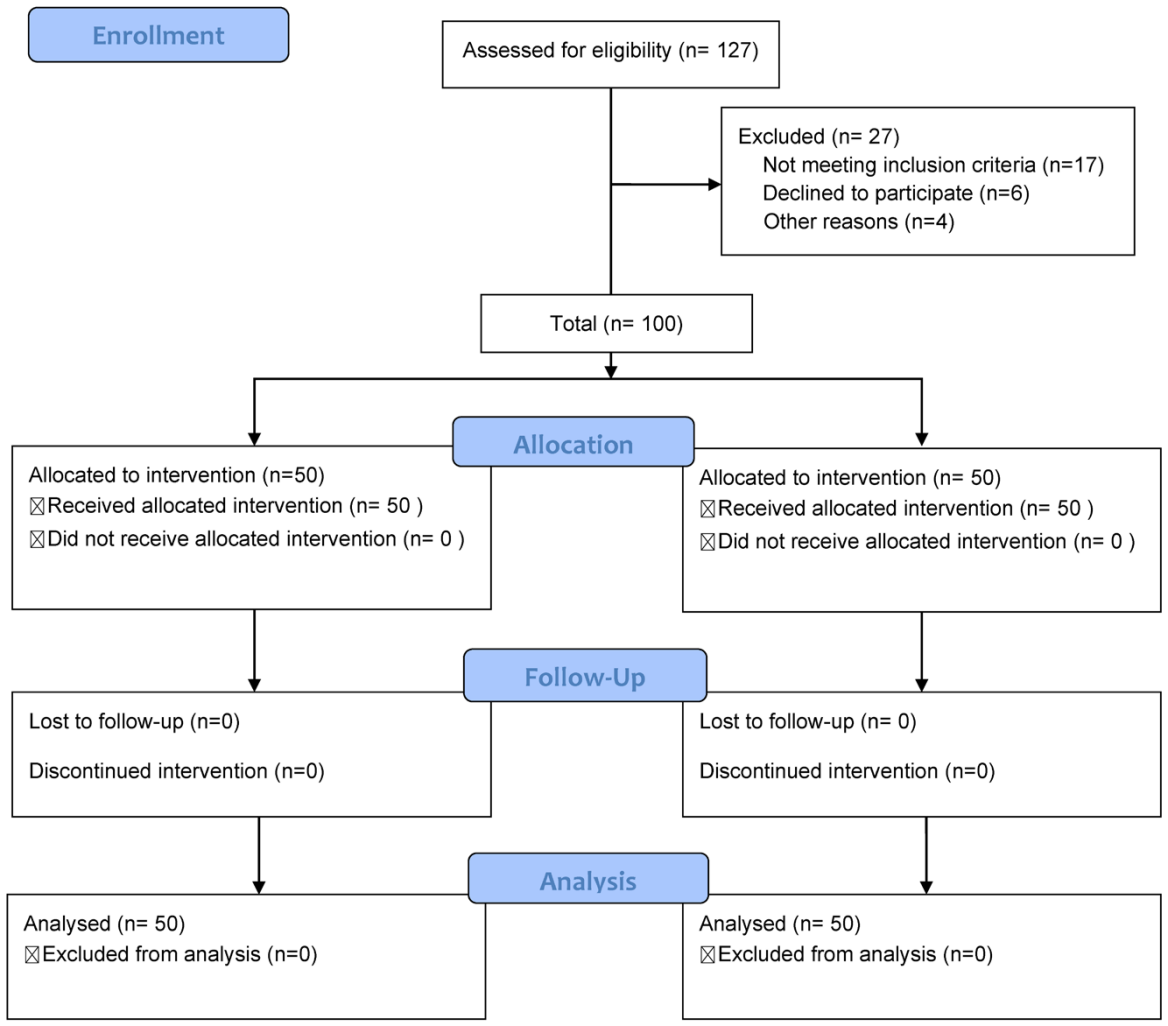
Flow diagram of the study design and population.

All the participants provided informed consent. The study protocol was rigorously reviewed and approved by the Ethics Committee of the Affiliated Hospital of Beijing Hua University (Fig. 1).

### 2.2. Therapeutic approach and surgical techniques for gastrointestinal reconstruction

The patients were positioned in a lithotomy stance and underwent successful intubation and anesthesia. A small incision below the navel was made to insert a Veress needle to establish pneumoperitoneum, 5 trocars were strategically placed: one 10-mm trocar at the umbilicus for the camera, 2 5-mm trocars in the right and left upper quadrants, and 2 additional 12-mm trocars in the right and left mid-clavicular lines for operative manipulation. This trocar configuration facilitated optimal access and maneuverability during the laparoscopic procedure. Subsequently, radical gastrectomy adhering to the D2 guidelines was performed for distal gastric cancer. For those who underwent the Billroth II procedure, the duodenal stump was securely closed following resection. A specific type of anastomotic stapler, designed for creating secure connections between sections of the gastrointestinal tract, was utilized to attach the jejunum to the gastric remnant. The anastomosis between the jejunum and gastric remnant was performed using an anti-peristaltic orientation. A 45mm linear stapler, specifically designed for gastrointestinal surgeries, was employed to create this anastomosis. This was followed by the use of reinforcement sutures at the anastomotic site. Alternatively, in the Uncut Roux-en-Y group, the gastrojejunostomy was executed in a peristaltic direction using a 60 mm linear stapler, ensuring alignment with the natural movement of the gastrointestinal tract. After the initial gastrojejunostomy, further incisions were made at distances of 10 cm and 25 cm to perform Braun anastomosis. Subsequently, the openings created for the anastomosis were securely closed. To strengthen the gastrojejunostomy site, additional sutures were applied approximately 5 cm away from the site to ensure stability and integrity of the anastomosis.

### 2.3. Evaluative criteria and observational parameters

Surgical Parameters: We compared surgical time, intraoperative blood loss, time to first flatus, time to first ambulation, time to oral intake, and length of hospital stay between the 2 patient groups.

Quality of Life Assessment: The Visick grading index used for postoperative quality of life evaluation classifies patients into 4 distinct categories. Grade I patients demonstrated exemplary postoperative recovery, reported no gastrointestinal issues, and maintained good nutritional status. In Grade II, patients experienced mild gastrointestinal symptoms such as bloating and diarrhea, and some manifested mild dumping syndrome or reflux esophagitis; however, these conditions were successfully managed through dietary modifications, and the patients’ nutritional status remained favorable. Grade III included patients who experienced mild-to-moderate symptoms of dumping syndrome or reflux esophagitis, which were alleviated through pharmacological interventions, although their nutritional status was only average. Lastly, Grade IV consisted of patients who exhibited moderate-to-severe gastrointestinal issues, including complications that significantly disrupted their quality of life. These symptoms were largely unresponsive to medication and poor nutritional status often necessitated further surgical intervention.

Postoperative Complications: An assessment of postoperative complications within a 6-month period was conducted for both groups. This included the incidence rates of infection, intestinal obstruction, dumping syndrome, bile reflux, anastomotic fistula, and reflux gastritis.

### 2.4. Statistical analysis

Statistical evaluations were performed using SPSS software version 27.0. Prior to analysis, all datasets underwent normality assessment and were classified as either quantitative or categorical variables. For quantitative variables that followed a Gaussian distribution, independent sample t-tests were used to ascertain intergroup statistical significance. The results for these variables are subsequently presented as arithmetic means and standard deviations (mean ± SD). Categorical variables were tabulated in terms of frequencies and relative percentages and subjected to chi-square (χ^2^) tests to scrutinize the relationships or independence among categories. All tests of hypothesis were 2-sided, and a significance level of *P* < .05 was considered the threshold for statistical relevance. This alpha level was selected in alignment with widely accepted scholarly criteria, thereby optimizing the balance between Type I and Type II errors.

## 3. Results

### 3.1. Participant analysis

In this study, 100 patients were included, adhering to specific inclusion and exclusion criteria. The patients were classified into 2 groups based on the type of anastomosis performed: uncut Roux-en-Y and Billroth II. Statistical analysis revealed that the 2 groups were comparable in terms of sex distribution, age, preoperative BMI, and TNM staging of gastric cancer. No statistically significant differences were found between these parameters (*P* > .05, Table 1).

**Table 1:** Comparison of demographic and clinical characteristics between patients undergoing uncut Roux-en-Y anastomosis and Billroth II anastomosis.

Criteria	Uncut Roux-en-Y group (N = 50)	Billroth II group (N = 50)	t/χ² value	*P* value
Sex (M/F)	30/20	28/22	0.28	>.05
Age (yr)	53.0 ± 9.8	54.5 ± 10.1	0.16	>.05
Pre-operative BMI (kg/m²)	24.5 ± 2.3	24.0 ± 2.0	0,68	>.05
TNM stage			0.26	>.05
Stage I	10	12	-	-
Stage II	25	24	-	-
Stage III	15	14	-	-

BMI = body mass index, M/F = male/female, TNM = tumor, node, metastasis.

### 3.2. Evaluation of surgical parameters between uncut Roux-en-Y and Billroth II techniques

In our investigation involving 100 patients, we aimed to discern the differences in various surgical parameters between 2 commonly employed techniques for gastric cancer resection: Uncut Roux-en-Y and Billroth II anastomosis. We observed that the surgical time in the Uncut Roux-en-Y group was significantly longer (*P* < .001). However, other *parameters*, including intraoperative blood loss, time to first flatus, time to first oral intake, time to first mobilization, and length of hospital stay, showed no statistically significant differences between the 2 groups (all *P* > .05). Our findings suggest that while the Uncut Roux-en-Y technique might require a longer operative time, both procedures appear to be equivalent in terms of other operative and postoperative parameters (Table 2).

**Table 2:** Comparative analysis of surgical parameters between uncut Roux-en-Y and Billroth II anastomosis groups.

Metrics	Uncut Roux-en-Y group (N = 50) (x-±s)	Billroth II group (N = 50) (x-±s)	t value	*P* value
Surgical time (min)	220.0 ± 21.9	203.4 ± 20.6	3.88	<.001
First flatus time (h)	19.6 ± 2.2	20.8 ± 2.4	1.28	.202
First oral intake (d)	4.42 ± 1.15	4.81 ± 1.31	0.21	.834
First time out of bed (d)	1.56 ± 0.52	1.68 ± 0.49	1.23	.22
Hospital stay (d)	8.90 ± 2.40	8.99 ± 2.12	0.33	.742
Intraoperative blood loss (mL)	73.1 ± 12.5	70.5 ± 13.2	0.78	.438

Note: In the table, “x-±s” denotes the mean (x) plus or minus the standard deviation (s) for each metric.

### 3.3. Assessment of patient-reported outcomes using Visick grading 6 months postoperatively

To evaluate the quality of life and symptom relief in patients 6 months after either Uncut Roux-en-Y or Billroth II anastomosis, we employed the Visick grading system. We found noteworthy differences in patient-reported outcomes between the 2 groups. Specifically, Grade I outcomes, which are indicative of optimal symptom relief and quality of life, were significantly more prevalent in the Uncut Roux-en-Y group (50.0%) than in the Billroth II group (26.0%). Conversely, Grade III, which indicates substantial postoperative complaints, was observed more frequently in the Billroth II group (30.0%) than in the Uncut Roux-en-Y group (8.0%, Table 3).

**Table 3:** Six-mo postoperative Visick grading in patients undergoing uncut Roux-en-Y vs Billroth II anastomosis.

Visick grade	Uncut Roux-en-Y group (N = 50) [n (%)]	Billroth II group (N = 50) [n (%)]	t value	*P* value
Grade I	25 (50.0)	13 (26.0)	3.88	<.01
Grade II	20 (40.0)	21 (42.0)	0.16	.51
Grade III	4 (8.0)	15 (30.0)	3.21	<.01
Grade IV	1 (2.0)	1 (2.0)	0.23	.32

Chi-square Test for Linear-by-Linear Association: χ² = 14.818, *P* = .0009995.

### 3.4. Evaluation of postoperative complications in uncut Roux-en-Y and Billroth II groups

The incidence and types of postoperative complications in the Uncut Roux-en-Y and Billroth II groups were meticulously assessed to understand their potential clinical implications. Notable variations were observed, particularly in the prevalence of the Dumping Syndrome and Bile Reflux. The Uncut Roux-en-Y group recorded a relatively lower incidence of Dumping Syndrome (2.0%) compared to a significantly higher rate of 16.0% in the Billroth II group, as indicated by a χ² value of 4.050 and a *P* value of .044. Likewise, Bile Reflux was more prevalent in the Billroth II group (26.0%) than in the Uncut Roux-en-Y group (4.0%), underlined by a χ² value of 7.100 and *P* value of .007. The data suggest that while both surgical procedures have inherent risks of postoperative complications, the Billroth II procedure may be associated with a higher likelihood of Dumping Syndrome and Bile Reflux (Table 4).

**Table 4:** Comparison of postoperative complications between patients undergoing uncut Roux-en-Y vs Billroth II anastomosis.

Complications	Uncut Roux-en-Y group (N = 50) [n (%)]	Billroth II group (N = 50) [n (%)]	χ² value	*P* value
Bile reflux	2 (4.0)	13 (26.0)	7.1	.007
Dumping syndrome	1 (2.0)	8 (16.0)	4.05	.024
Reflux gastritis	1 (2.0)	4 (8.0)	1.475	.22
Bowel obstruction	1 (2.0)	3 (6.0)	0.765	.38
Anastomotic fistula	2 (4.0)	3 (6.0)	0.085	.77
Infection	2 (4.0)	3 (6.0)	0.035	.78

## 4. Discussion

The prevalence of minimally invasive surgical interventions for gastric cancer, such as laparoscopic gastrectomy, has increased in response to the growing patient demand and technological advancements.^[10,11]^ This alteration often leads to postoperative complications that significantly affect the patients’ quality of life and psychological well-being. Thus, the focus has shifted towards various methods of digestive tract reconstruction to mitigate these complications postoperatively.

Among the commonly adopted techniques for gastrointestinal reconstruction are Billroth I, Billroth II, and Roux-en-Y anastomoses, including their modified form, Uncut Roux-en-Y.^[12,13]^ Despite numerous studies, there is a lack of consensus among clinicians and researchers regarding the most suitable method for digestive tract reconstruction. Billroth II anastomosis, for instance, is straightforward, requires less time to perform, and is easier for surgical teams to master, making it widely applicable.^[14]^ It also circumvents the issue of excessive tension seen in Billroth I anastomosis, which is usually performed between the remnant stomach and the more pliable jejunum. However, its key drawback is the alteration of the natural pathway for food, which may result in postoperative complications such as dumping syndrome, bile reflux, and reflux gastritis.^[15]^

In contrast, our study demonstrated that patients in the Uncut Roux-en-Y group had longer surgical time than those in the Billroth II anastomosis group. Nevertheless, no statistically significant difference in intraoperative blood loss was found between the 2 groups. This observation is likely due to the fact that major bleeding events during gastrectomy are primarily associated with gastric mobilization and lymph node dissection, as opposed to the digestive tract reconstruction phase. Our statistical analysis further showed no marked differences in clinical endpoints, such as time to first flatus, time to ambulation, time to oral feeding, and length of hospital stay (*P* > .05). A noteworthy finding of our study was the improved Visick grading in the Uncut Roux-en-Y group at the 6-month postoperative mark, which was statistically significant when compared to the Billroth II group (*P* < .01). Additionally, the Uncut Roux-en-Y procedure resulted in significantly lower incidence rates of dumping syndrome and bile reflux (*P* < .05).

The Uncut Roux-en-Y anastomosis can be conceptualized as an enhanced version of Billroth II, augmented by the addition of the Braun anastomosis and jejunal loop ligation. This innovative approach serves a dual purpose: it maintains the jejunum anatomical and functional continuity while concurrently mitigating bile reflux into the remnant stomach.^[16,17]^ Although the Uncut Roux-en-Y technique alters the physiological architecture of the digestive tract, our data suggest that it does not adversely affect neuromuscular electrophysiology. Importantly, this advanced form of anastomosis appears to successfully mitigate complications commonly associated with both Billroth II and standard Roux-en-Y, including dumping syndrome, bile reflux, blind loop syndrome, functional gastric outlet delay, and Roux stasis syndrome.^[18]^ The Visick grading at 6 months postoperatively shows a statistically significant improvement in the Uncut Roux-en-Y group compared to the Billroth II group (*P* < .01). Furthermore, the incidence of dumping syndrome and bile reflux was significantly lower in the Uncut Roux-en-Y group (*P* < .05), as evidenced by our meticulous assessment of postoperative complications. These findings provide objective evidence supporting the benefits of the Uncut Roux-en-Y anastomosis in reducing specific postoperative complications and enhancing patient quality of life.

Our study has some limitations that warrant discussion. First, the sample size was relatively small, which may reduce the power of the statistical analyses and potentially limit the generalizability of our findings. Second, our study was a single-center investigation, and the results might differ in multicenter settings or across diverse populations. Third, the follow-up duration was only 6 months, which is insufficient for evaluating long-term outcomes and late-onset complications. Fourth, the surgeons’ expertise and techniques could introduce a level of bias, as the surgeons performing the Uncut Roux-en-Y procedure were specialized in this particular technique. Lastly, we did not incorporate a comprehensive quality-of-life assessment, including nutritional status, psychological well-being, and other subjective parameters, which might offer a more holistic view of the postoperative outcomes. Future research should address these shortcomings to provide more robust evidence to support the efficacy and safety of Uncut Roux-en-Y anastomosis.

## 5. Conclusions

In conclusion, the utilization of Uncut Roux-en-Y anastomosis for digestive tract reconstruction following laparoscopic D2 gastrectomy for distal gastric cancer demonstrates a distinct advantage over the Billroth II procedure. Although the surgery time for the Uncut Roux-en-Y approach was comparatively longer, it yielded significant improvements in patient quality of life. Our findings show that this advanced technique effectively reduced the incidence of bile reflux and dumping syndrome.

## Acknowledgments

We thank all participants for their hard work.

## Author contributions

**Conceptualization:** Bufei Zhao, Ting Hu.

**Data curation:** Zhun Yu.

**Formal analysis:** Zhun Yu.

**Investigation:** Zhun Yu.

**Methodology:** Bufei Zhao, Ting Hu.

**Resources:** Bufei Zhao, Ting Hu.

**Software:** Zhun Yu.

**Writing – original draft:** Bufei Zhao.

**Writing – review & editing:** Bufei Zhao.

## References

[1] Smyth EC Nilsson M Grabsch HI. Gastric cancer. Lancet. 2020;396:635–48. 32861308 10.1016/S0140-6736(20)31288-5

[2] Karimi P Islami F Anandasabapathy S. Gastric cancer: descriptive epidemiology, risk factors, screening, and prevention. Cancer Epidemiol Biomarkers Prev. 2014;23:700–13. 24618998 10.1158/1055-9965.EPI-13-1057 PMC4019373

[3] Chen QY Xie JW Zhong Q. Safety and efficacy of indocyanine green tracer-guided lymph node dissection during laparoscopic radical gastrectomy in patients with gastric cancer: a randomized clinical trial. JAMA Surg. 2020;155:300–11. 32101269 10.1001/jamasurg.2019.6033

[4] Huang C Liu H Hu Y. Laparoscopic vs open distal gastrectomy for locally advanced gastric cancer: five-year outcomes from the CLASS-01 randomized clinical trial. JAMA Surg. 2022;157:9–17. 34668963 10.1001/jamasurg.2021.5104 PMC8529527

[5] Nishizaki D Ganeko R Hoshino N. Roux-en-Y versus Billroth-I reconstruction after distal gastrectomy for gastric cancer. Cochrane Database Syst Rev. 2021;9:CD012998. 34523717 10.1002/14651858.CD012998.pub2 PMC8441595

[6] Rosa F Quero G Fiorillo C. Billroth II reconstruction in gastric cancer surgery: a good option for Western patients. Am J Surg. 2019;218:940–5. 30894253 10.1016/j.amjsurg.2019.03.009

[7] Chen K Xu X Mou Y. Totally laparoscopic distal gastrectomy with D2 lymphadenectomy and Billroth II gastrojejunostomy for gastric cancer: short- and medium-term results of 139 consecutive cases from a single institution. Int J Med Sci. 2013;10:1462–70. 24046519 10.7150/ijms.6632 PMC3775102

[8] Imai Y Lee SW Sakaguchi S. Comparison of the gastric microbiome in Billroth I and Roux-en-Y reconstructions after distal gastrectomy. Sci Rep. 2022;12:10594. 35732881 10.1038/s41598-022-14886-4 PMC9217802

[9] Wu CH Huang KH Chen MH. Comparison of the long-term outcome between Billroth-I and Roux-en-Y reconstruction following distal gastrectomy for gastric cancer. J Gastrointest Surg. 2021;25:1955–61. 33205309 10.1007/s11605-020-04867-1

[10] Ang TL Fock KM. Clinical epidemiology of gastric cancer. Singapore Med J. 2014;55:621–8. 25630323 10.11622/smedj.2014174 PMC4291998

[11] Fock KM. Review article: the epidemiology and prevention of gastric cancer. Aliment Pharmacol Ther. 2014;40:250–60. 24912650 10.1111/apt.12814

[12] Tran TB Worhunsky DJ Squires MH. To Roux or not to Roux: a comparison between Roux-en-Y and Billroth II reconstruction following partial gastrectomy for gastric cancer. Gastric Cancer. 2016;19:994–1001. 26400843 10.1007/s10120-015-0547-3

[13] Sah BK Chen MM Yan M. Gastric cancer surgery: Billroth I or Billroth II for distal gastrectomy? BMC Cancer. 2009;9:428. 20003202 10.1186/1471-2407-9-428 PMC2794879

[14] Yang D He L Tong WH. Randomized controlled trial of uncut Roux-en-Y vs Billroth II reconstruction after distal gastrectomy for gastric cancer: which technique is better for avoiding biliary reflux and gastritis? World J Gastroenterol. 2017;23:6350–6. 28974902 10.3748/wjg.v23.i34.6350 PMC5603502

[15] Yang K Zhang W Chen Z. Comparison of long-term quality of life between Billroth-I and Roux-en-Y anastomosis after distal gastrectomy for gastric cancer: a randomized controlled trial. Chin Med J (Engl). 2023;136:1074–81. 37014767 10.1097/CM9.0000000000002602 PMC10228481

[16] Li Y Wang Q Yang KL. Uncut Roux-en-Y might reduce the rate of reflux gastritis after radical distal gastrectomy: an evidence mapping from a systematic review. Int J Surg. 2022;97:106184. 34861427 10.1016/j.ijsu.2021.106184

[17] Chen Y Zheng T Chen Y. Totally laparoscopic total gastrectomy with Uncut Roux-en-Y for gastric cancer may improve prognosis: a propensity score matching comparative study. Front Oncol. 2022;12:1086966. 36620551 10.3389/fonc.2022.1086966 PMC9822261

[18] Huang Y Wang S Shi Y. Uncut Roux-en-Y reconstruction after distal gastrectomy for gastric cancer. Expert Rev Gastroenterol Hepatol. 2016;10:1341–7. 27748146 10.1080/17474124.2016.1248404

